# Visual Acuity and Retinal Disease

**Published:** 1907-09-21

**Authors:** 


### The Hospital

A Journal of

The Medical Sciences and Hospital Administration.

New Series. No. 26, Vol. I. [No. 1098, Vol. XLII.] SATURDAY, SEPTEMBER 21, 1907.

### VISUAL ACUITY AND RETINAL DISEASE.

There are many reasons for believing that the general body of the profession is coming, to a fuller and fuller extent, to appreciate the contention that to allow certain departments of practice to drift entirely into the hands of specialists is a profound mistake. The numerous post-graduation opportunities offered in these departments by the medical schools and hospitals are now largely taken advantage of, and with benefit, we are convinced, to all concerned. It has become evident that a comparatively short clinical experience, when seconded by diligent application and study, is sufficient to afford a basis on which may be readily constructed a knowledge and skill adequate to deal successfully with the great majority of the local disturbances now largely referred to the specialist. In all this we can see nothing but good to the efficiency of the medical profession and to the public welfare. Among the results will certainly be an increase in the respect and regard paid both to the individual practitioner and the profession generally, as also an improved appreciation of the relation between local lesions and general constitutional disturbances. Prominent in this association is a much extended cultivation of the use of the ophthalmoscope. Even yet, however, the value of this instrument to the general practitioner is but inadequately appreciated, though, it may be hoped, the date is not far distant at which it may take rank as an agent not less necessary than the stethoscope and the clinical thermometer.

One of the directions in which the ophthalmoscope has a special field of opportunity is provided by the fact that structural disease, and even extensive structural disease, may be present in the fundus of the eye without any appreciable defect of vision. Moreover, this remark applies not only to the subjective sensations of the patient, but also to the usual standard of vision as determined by the use of the test types. It cannot be too frequently repeated that a capacity to read correctly both the near and distant types is quite consistent with the existence of gross disease in the structure of the retina. This truth, in respect to the existence of double optic neuritis, was announced to the profession many years ago by Dr. Hughlings Jackson in connection with the diagnosis of intra-cranial tumour. Yet it may be doubted whether even now the importance of the statement is fully appreciated. A patient with more or less headache, and perhaps no other symptom, is regarded as the victim of some vague functional disturbance; no examination is made of the fundus oculi because no complaint of defective vision is proffered. Only perhaps after weeks or months is the necessary investigation applied, and then the discovery of optic neuritis changes in a moment both the diagnostic position and the prognostic outlook. How frequently this experience repeats itself it is, of course, impossible to say. It is sufficient to affirm that it occurs, and that its occurrence is mainly due to a defective appreciation of the statement that optic neuritis may exist even though the patient has a full standard of visual acuity.

But optic neuritis does not stand alone in this respect. Retinitis and retinal hæmorrhages must each be placed with it in the same category. It is not a rare experience to find that a patient in whom the ophthalmoscope shows even an extensive development of albuminuric retinitis neither makes complaint of visual defect nor falls below the ordinary standard of the test types. Exactly the same is true of multiple small hæmorrhages occurring, perhaps, in chronic intestinal nephritis. This disease, as is well known, is of very insidious onset, and is apt for some considerable time to be overlooked. If the patient, as is common, makes no complaint of failing sight the eyes are commonly not examined. Yet in a certain percentage of the cases such an examination would reveal facts, in the shape either of retinitis or hæmorrhage, which would at once make the practitioner alive to the situation; and the importance of such an observation becomes all the greater when it is remembered that in the disease in question, one of the most suggestive symptoms—namely, albuminuria, may be absent even to repeated investigation. In short, forgetfulness of the fact that good sight does not always mean a healthy retina causes the early diagnosis of some cases of chronic renal disease to be missed.

In speaking of retinal hæmorrhages as existing without prejudice to vision, it is essential in this relation to mention the subject of pernicious anæmia. Here is a disease which often presents a difficult problem for diagnosis. In its more acute forms the high temperatures are very apt to prove misleading, whilst in less urgent cases the symptoms may readily receive inadequate appreciation. Complaint of defective vision is uncommon, yet ophthalmoscopic examination will frequently show multiple retinal hæmorrhages, and in the circumstances these can hardly fail to indicate the diagnosis. Many similar illustrations might be quoted, but enough has been advanced to illustrate how important is the proposition that effective vision is not inconsistent with gross retinal disease.

